# Transdermal Delivery of an Adjuvanted SARS-CoV-2 Triple RBD Subunit Vaccine in Mice Using Dissolving Microneedle Array Patch

**DOI:** 10.3390/pharmaceutics18101236

**Published:** 2026-09-29

**Authors:** Eun Kim, Moon-Su Lee, Muhammad S. Khan, Shaohua Huang, Joshua Lee, Dohyeon Jeong, Andrea Gambotto

**Affiliations:** 1 Department of Surgery, School of Medicine, University of Pittsburgh, Pittsburgh, PA 15213, USA; kime@upmc.edu (E.K.);; 2 Medical Business Division, Raphas Co., Ltd., Seoul 07793, Republic of Korea; 3 Department of Infectious Diseases and Microbiology, School of Public Health, University of Pittsburgh, Pittsburgh, PA 15261, USA; 4 Division of Infectious Diseases, Department of Medicine, School of Medicine, University of Pittsburgh, Pittsburgh, PA 15261, USA; 5 UPMC Hillman Cancer Center, Pittsburgh, PA 15232, USA

**Keywords:** COVID-19, SARS-CoV-2, microneedle array patch, triple RBD, recombinant protein subunit, Squalene-based oil-in-water emulsion adjuvant, humoral immunity

## Abstract

**Background**: The continuous emergence of antigenically distinct SARS-CoV-2 variants of concern (VOCs) necessitates the development of versatile and effective vaccine platforms capable of inducing broad and sustained immunity. **Methods**: We designed a chimeric trivalent recombinant protein, TriRBD, incorporating the receptor-binding domains (RBDs) of the Delta, Omicron BA.1, and XBB.1 subvariants. To enhance delivery efficiency and immunogenicity, TriRBD was formulated into a dissolving hyaluronic-acid-based microneedle array patch (MAP) using the droplet extension (DEN) technique. We investigated the synergistic effects of two squalene-based oil-in-water (O/W) emulsions, SqTS (Squalene/Tween-80/Span-85) and SqT (Squalene/Tween-80), as adjuvants within the MAP after gamma-irradiation sterilization, alongside safety evaluations via rabbit skin tests. **Results**: The SqTS-adjuvanted MAP formulation was found to be non-irritating. Immunogenicity studies in BALB/c mice revealed that MAP-delivered TriRBD elicited significantly higher and more durable IgG endpoint titers (EPT) compared to intramuscular injection, with robust antibody levels sustained for up to 52 weeks. Notably, the irradiated SqT-adjuvanted MAP group exhibited a highly balanced Th2/Th1 immune response (IgG1/IgG2a ratio), while the irradiated SqTS-adjuvanted MAP group shifted toward a Th2-preferred against TriRBD. Although the EPT against the Omicron BA.1 variant was relatively lower, the irradiated MAP-SqT-TriRBD group elicited significantly both of IgG1 and IgG2a, resulting in a balanced and broadened antibody repertoire. **Conclusions**: These findings demonstrate that combining a trivalent RBD antigen with a SqT-adjuvanted MAP platform provides a potent, safe, and thermostable strategy for broad-spectrum protection against evolving SARS-CoV-2 variants and future pandemic threats.

## 1. Introduction

Vaccination remains the most effective public health strategy for controlling the COVID-19 pandemic, significantly reducing hospitalizations, severe disease, and mortality rates [1]. Despite the rapid development and deployment of various vaccines, new infections continue to escalate periodically worldwide. This persistence is primarily driven by the emergence of antigenically distinct SARS-CoV-2 variants of concern (VOCs), which can evade existing immunity. Consequently, there is an urgent need for innovative vaccine format capable of preventing the infection and transmission of diverse VOCs to mitigate future pandemic waves.

The spike protein on the SARS-CoV-2 virus surface mediates viral attachment and entry through its receptor-binding domain (RBD), making the RBD a primary target for vaccine design [2]. However, the protective efficacy of traditional RBD-based vaccines has declined due to emerging mutations. To broaden the spectrum of the immune response, chimeric strategies utilizing RBD dimer or trimers—constructed by tandemly linking RBDs from different strains—have been developed [3,4,5]. Notably, recent studies show that conjugating these RBD-based immunogens with the human IgG Fc fragment or formulating them with the mucosal adjuvant flagellin KFD successfully elicits robust neutralizing activity and coordinated mucosal IgA responses against Omicron variants [6,7].

Microneedle array patches (MAPs) have emerged as a promising delivery platform due to their ability to target the immune-rich environment of the skin. We previously reported that the intradermal delivery of the SARS-CoV-2 S1 protein via MAPs is both effective and long-lasting [8,9]. To further enhance the magnitude, breadth, and durability of these immune responses including enabling dose sparing, the incorporation of adjuvant into MAP is essential. Various adjuvants, including cholera toxin, CpG oligonucleotide, Monophosphoryl lipid A, poly(I:C), and saponins, have been explored in preclinical MAP studies for diseases such as hepatitis B, Human immunodeficiency virus (HIV), Ebola, and influenza [10]. While aluminum-type adjuvants have also been investigated for MAP delivery [10,11,12], their efficacy remains controversial; a study reported that aluminum-type adjuvant did not induce high efficacy due to the low T-cell-mediated immune responses [13], while others observed enhanced immunity compared to non-adjuvanted formulations [14]. Recently, cyclic dinucleotides (cGMP) and saponin-based system (cGMP/Saponin) have shown promise in dissolving microneedle array patches (MAP) for influenza vaccines in aged mice [15,16].

In this study, we focused on squalene-based oil-in-water (O/W) emulsion adjuvants for MAP delivery. Squalene emulsions, such as AddaVax™, are known to stimulate both Th1 and Th2 immune responses by activating antigen-presenting cells (APCs) to prime CD4+ and CD8+ T cells [17,18]. MF59- and MF59-like emulsions have been successfully utilized in various influenza and recombinant protein-based COVID-19 vaccines [19,20,21,22]. Here, we designed a chimeric trivalent RBD recombinant protein (TriRBD) incorporating RBDs from the Delta, Omicron BA.1, and XBB.1 subvariants. Furthermore, to ensure the clinical safety and biocompatibility of the adjuvanted platform, we conducted a comprehensive in vivo skin irritation test using a rabbit model. We assessed the immunogenicity of TriRBD delivered via both irradiated and non-irradiated MAPs formulated with two types of squalene-based O/W emulsions (SqTS or SqT). Our findings demonstrated that irradiated MAP delivery of TriRBD adjuvanted with SqT elicits a balanced, broad-spectrum immune responses that surpasses intramuscular administration. These findings establish SqT as an ideal adjuvant for MAP-based protein subunit vaccine delivery, offering a potent strategy for future pandemic preparedness.

## 2. Materials and Methods

### 2.1. Antigens

The trivalent receptor-binding domain antigen (TriRBD) was designed to comprise the receptor-binding domains (RBDs, R319-K537 from the spike protein) of SARS-CoV-2 XBB.1.5 (GenBank accession no. WGO53701), BA.5 (GenBank accession no. WJF72330), and Delta (B.1.617.2) (GenBank accession no. QYM88595), arranged sequentially and separated by flexible glycine linkers (GGGGS)_3_. The TriRBD construct was fused at the C terminus to the Toll-like receptor 4 (TLR4) agonist peptide RS09 (APPHALS), followed by a C-tag (EPEA). To generate the pAd/TriRBD plasmid, the TriRBD coding sequence was codon-optimized for mammalian expression using UpGene algorithm and subsequently cloned into the shuttle vector pAdlox (GenBank accession no. U62024) via *Sal* I and *Not* I restriction sites [23,24]. The integrity of the resulting plasmid was confirmed by DNA sequencing. The schematic 3D structure of TriRBD recombinant protein was predicted using AlphaFold 3 [25]. Secondary structure predictions were performed using the Discrimination of Secondary Structure Class (DSC) method via the Network Protein Sequence Analysis (NPSA) server [26,27].

Recombinant TriRBD protein was produced by transient transfection of Expi293 cells (Thermo Fisher Scientific, Waltham, MA, USA) with pAd/TriRBD using the ExpiFectamie^TM^ 293 Transfection Kit (Thermo Fisher Scientific, Waltham, MA, USA) as described previously [8,28,29]. Secreted proteins were purified using a CaptureSelect^TM^ C-tagXL Affinity column (Thermo Fisher Scientific, Waltham, MA, USA) according to the manufacturer’s instruction. Protein concentration was determined by the bicinchoninic acid (BCA) assay kit (Thermo Fisher Scientific, Waltham, MA, USA) using bovine serum albumin (BSA) as the standard. Protein integrity and purity were accessed by reducing SDS-PAGE followed by silver staining.

### 2.2. SDS-PAGE and Western Blot

Purified proteins were analyzed by SDS-PAGE followed by silver staining and Western blot. Briefly, protein samples were mixed with Laemmli sample buffer containing 2% SDS with beta-mercaptoethanol (β-ME), boiled, and separated on 10% Tris-Glycine SDS-PAGE gels. The proteins were then transferred onto nitrocellulose membranes. The membranes were blocked for 1 h at room temperature (RT) with 5% nonfat milk in TBS-T and incubated overnight at 4 °C with a biotinylated anti-C-tag monoclonal antibody (1:4000, Thermo Fisher Scientific, Waltham, MA, USA). After washing, the membranes were incubated for 1 h at RT with horseradish peroxidase (HRP)-conjugated streptavidin (1:4000, Bio-Rad, Hercules, CA, USA). Immunoreactive signals were detected using enhanced chemiluminescence (ECL) substrate reagents and imaged an iBright 1500 system (Thermo Fisher Scientific, Waltham, MA, USA).

### 2.3. Preparation of Adjuvant

SqTS (5.0% squalene, 0.5% Tween-80, 0.5% Span-85) and SqT (5.0% squalene, 1.0% Tween-80) were prepared in 10 mM sodium citrate buffer, followed by homogenization at 12,000 psi using a Microfluidizer (M-110L, Microfluidics, Middleborough, MA, USA) (Supplementary Figure S1A). The resulting emulsions were sterilized by passage through a with 0.22 μm polysulfone filter (Gelman Sciences, Ann Arbor, MI, USA) and stored at 4 °C. The mean particle size and polydispersity index (PDI) of the emulsion droplets were measured by dynamic light scattering (DLS) using a Zetasizer Nano ZS (Malvern Instruments Ltd., Malvern, UK). All emulsions were vortexed and appropriately diluted with deionized water before measurement.

### 2.4. Preparation of Dissolving Microneedle Array Patches

Dissolving microneedle array patches (MAPs) containing recombinant proteins were fabricated using the droplet extension (DEN) technique. In this method, dispensed droplets were extended and solidified into cone-shaped microstructures by controlled air blowing, as described previously [8,30]. The MAPs used in this study were provided by Raphas Co., Ltd. (Seoul, Republic of Korea). Briefly, a 5 × 5 base arrays (1.76 cm^2^) was fabricated onto a circle patterned hydrocolloid adhesive sheet (Hiks C&T, Seongnam-si, Republic of Korea) (2.0 cm^2^) using a customized MPP-1 dispenser (Musashi Engineering, Inc., Tokyo, Japan) with 10% (*w*/*v*) Carboxyl methyl cellulose (CMC, Ashland Inc., Tokyo, Japan). The dispensed array was then dried overnight at RT. Subsequently, a viscous solution containing antigens and/or adjuvant formulated with Hyaluronic acid (HA, Contipro Inc., Dolní Dobrouč, Czech Republic) was dispensed onto the base array. The two dispensed droplets were brought into contact and extended to the target length, followed by symmetric air blow at RT to solidify the extended droplets, resulting in cone-shaped microstructures.

The mechanical strength of each MAP was measured using a universal testing machine (UTM, ZwickRoell, Ulm, Germany). Briefly, a MAP was mounted on the UTM stage and aligned with the probe, which was advanced vertically until microneedle fracture occurred. The force required to fracture the microneedles was recorded as the mechanical strength. To support translational development toward clinical production of TriRBD, a subset of MAPs was sterilized by gamma irradiation to evaluate clinically relevant sterilization conditions and their effects on immunogenicity. Gamma irradiation was performed using a cobalt-60 irradiator (JS-8900, Nordion Inc., Ottawa, ON, Canada) at doses of 15~30 kGy.

### 2.5. Primary Skin Irritation Assay

To evaluate skin irritation caused by MAP application with/without SqTS emulsion adjuvant, six adult New Zealand white albino rabbits (2.5–3.5 kg) were used for the experiment. Four patches were tested on each rabbit, as follows: two MAP-SqTS patches and two empty MAPs (negative control). The animals were acclimated for at least 5 days before testing and housed individually in temperature-controlled (20–25 °C) and humidity-monitored (45–65%) conditions. The dorsal skin of each rabbit (11 × 18 cm) was shaved, and four test sites (two on the right side and two on the left), each measuring 1 × 1 inch, were marked. The two upper test sites were abraded to the stratum corneum using a sterile needle. To avoid the use of topical anesthetics, abrasion was performed to a depth sufficient to penetrate the epidermis without inducing bleeding. The two lower test sites were left unabraded. MAPs were applied to the shaved dorsal skin using a handheld spring applicator and secured by finger pressure for 10 s. The entire dorsal area was then wrapped with rubberized cloth and micropore tape for 24 h. Erythema and edema were evaluated according to the Draize scoring method (Table 1) [31,32]. Signs of erythema and eschar formation were scored on a scale of 0 to 4: 0 (no erythema), 1 (very slight erythema; barely perceptible), 2 (well-defined erythema), 3 (moderate-to-severe erythema), or 4 (severe erythema; beet redness to slight eschar formations; injuries in depth). Edema formation was scored as follows: 0 (no edema), 1 (very slight edema; barely perceptible), 2 (slight edema; edges of area well defined by definite raising), 3 (moderate erythema; raised approximately 1 mm), or 4 (severe edema; raised >1 mm and extending beyond the exposed area). Scores were recorded for both abraded and intact skin sites and were reassessed 72 h after MAP removal. The primary dermal irritation index (PII) was calculated by summing the average erythema and edema scores across all observation time points and dividing by the total number of observation periods [33,34]. The degree of irritation was classified based on the PDII values as follows: nonirritating (PDII = 0–0.5), slightly irritating (PDII ≥ 0.5–2.0), moderately irritating (PDII ≥ 2.0–5.0), or severely irritating (PDII ≥ 5.0–8.0).

### 2.6. Animals and Immunization

Female BALB/c mice (n = 5 animals per group) were bled from the retro-orbital vein before immunization. For intramuscular vaccination, the mice were primed with 5 μg of TriRBD formulated with SqTS (1:1, *v*/*v*) and injected into the thigh muscle. For microneedle-based vaccination, MAPs loaded with 5 μg of TriRBD, with (+) or without (−) gamma irradiation, were applied to the depilated dorsal skin of the back region, as described previously [9]. MAP formulations containing TriRBD with SqTS or SqT were referred to as MAP-SqTS-TriRBD and MAP-SqT-TriRBD, respectively. Prior to vaccination, hair at the vaccination site was removed by shaving and depilatory cream. MAPs were applied to the dehaired back skin of the mice using a handheld spring applicator and held in place by finger pressure for 10 s, followed by leaving it on the skin for more than 2 h. No adverse skin effects, such as skin irritation at the vaccinated region, were observed. At 3 weeks after primary immunization, the mice received booster immunizations with homologous immunogens. Blood samples were collected from the retro-orbital vein at intervals of 3 to 10 weeks through week 52. Serum samples were isolated, diluted, and used to evaluate TriRBD-, S1WU-, and S1OM-specific antibody responses by enzyme-linked immunosorbent assay (ELISA). Mice were maintained under specific pathogen-free conditions at the University of Pittsburgh. All experiments were conducted in accordance with institutional guidelines and were approved by the University of Pittsburgh Institutional Animal Care and Use Committee (IACUC; protocol number 23052950).

### 2.7. Assessment of Serum Humoral Antibodies

To investigate the immunogenicity of SARS-CoV-2 TriRBD recombinant proteins, ELISA was performed as previously described [8,9,28] Sera samples were collected from all mice before immunization (week 0) and at weeks of 3, 6, 9, 16, 26, 40, and 52 after immunization and analyzed for TriRBD-specific IgG, IgG1, and IgG2a antibodies. In addition, sera collected at weeks 0, 9, 26, and 52 were analyzed for SARS-CoV-2 S1OM-specific IgG, IgG1, and IgG2a response, and sera collected at week 16 were analyzed for S1WU-specific IgG responses. Briefly, ELISA plates were coated overnight at 4 °C with 200 ng per well of recombinant TriRBD, SARS-CoV-2 S1WU protein (Sino Biological, Beijing, China), or S1OM protein [28] in carbonate coating buffer (pH 9.5). The plates were then blocked with PBS containing 0.05% Tween 20 (PBS-T) and 2% bovine serum albumin (BSA) for 1 h at room temperature. The serum samples were serially diluted in PBS-T with 1% BSA and incubated overnight. After washing, horseradish peroxidase (HRP)-conjugated anti-mouse IgG, IgG1, or IgG2a (1:10,000, Jackson ImmunoResearch, West Grove, PA, USA) was added and incubated for 1 h. The plates were washed three times, developed with 3,3′5,5′-tetramethylbenzidine (TMB), and the reaction was stopped by adding 0.18 M sulfuric acid. Absorbance at 450 nm was measured using a SpectraMax iD5 microplate reader (Molecular Devices, San Jose, CA, USA).

### 2.8. Statistical Analysis

Statistical analyses were performed using GraphPad Prism (version 11, GraphPad Software, San Diego, CA, USA). Antibody endpoint titers were log-transformed prior to statistical analysis to satisfy normality assumptions required for geometric mean calculations and parametric testing. Statistical differences among multiple experimental groups at specific time point or comparisons within specified antigen groups across time points were evaluated using one-way analysis of variance (ANOVA) followed by Tukey’s multiple comparisons test to adjust for multiple pairwise comparisons (family-wise error rate α= 0.05). Data are presented as the means ± standard errors of the mean (SEM) or geometric means ± geometric standard deviation. Statistical significance is denoted as follows: * *p* < 0.05, ** *p* < 0.01, *** *p* < 0.001, **** *p* < 0.0001.

## 3. Results

### 3.1. Purification and Characterization of Recombinant TriRBD Proteins

**Figure 1:**
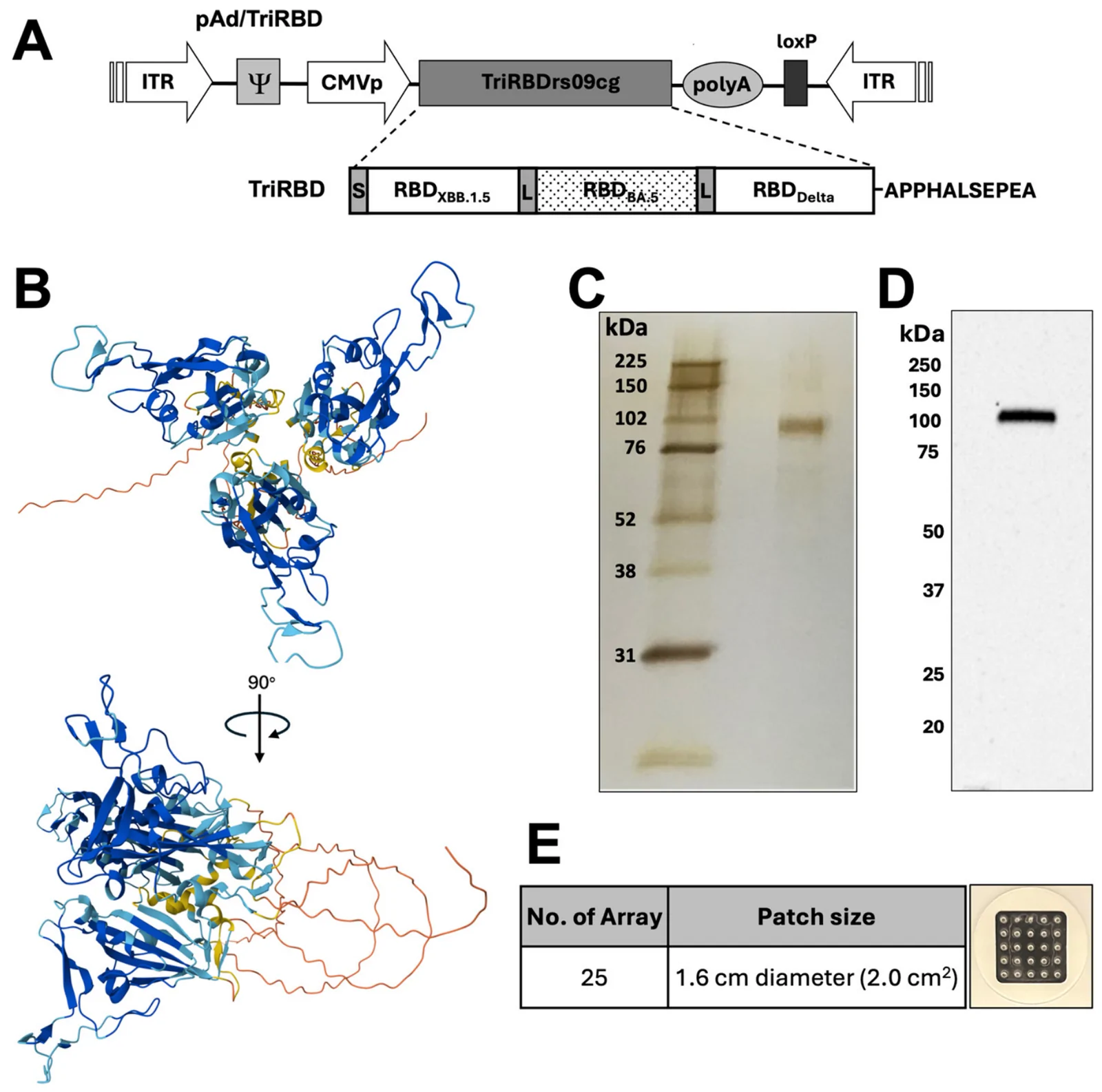
Construction and expression of recombinant SARS-CoV-2 TriRBD proteins. (**A**) A shuttle vector carrying the codon-optimized SARS-CoV-2 TriRBD gene encoding XBB.1.5 RBD, BA.5 RBD, and Delta (B.1.617.2) RBD with toll-like receptor 4 (TLR4) agonist peptide RS09 (APPHALS) and c-tag (EPEA) was designated as shown in the diagram. ITR: inverted terminal repeat; CMVp, cytomegalovirus promoter; RBD: receptor binding domain. (**B**) The schematic 3D structure of TriRBD was predicted by AlphaFold 3. (**C**) Purified TriRBD proteins isolated by c-tag affinity purification were separated by SDS-PAGE and visualized by silver staining. Molecular weight marker is indicated on the left. (**D**) Detection of the Purified TriRBD proteins by western blot using biotinylated anti-Ctag monoclonal antibody. (**E**) Fabrication of dissolving microneedle patch, MAP-TriRBD consisting of 25 microneedles in a 5 × 5 arrays (1.76 cm^2^) mounted on a circular hydrocolloid adhesive sheet (2.0 cm^2^).

The recombinant protein TriRBD for microneedle array patch fabrication was designed to include three sequential RBDs (XBB.1.5, BA.5, and Delta [B.1.617.2]) linked by a (GGGGS)_3_. To enhance immunogenicity and facilitate purification, a Toll-like receptor 4 (TLR4) agonist peptide RS09 (APPHALS) and a C-tag (EPEA) were fused to the C-terminal end. The sequence was codon-optimized for mammalian expression using UpGene algorithm [23,24] and subcloned into the shuttle vector pAdlox at Sal I and Not I sites to generate pAd/TriRBD (Figure 1A).

The predicted structure of TriRBD is presented in Figure 1B. Three heterologous RBDs (residues 319–537) derived from SARS-CoV-2 XBB.1.5, BA.5, and B.1.617.2 spike proteins were joined by flexible linkers to form a single-chain construct that co-assembled into a trimeric architecture (TriRBD). Secondary structure prediction indicated that the recombinant TriRBD protein adopted a proper fold consisting of 11.11% α-helix, 20.37% β-sheet, and 68.52% random coil. In comparison, individual monomeric RBDs predicted by DSC comprised 13.70% α-helix, 13.70% β-sheet, and 72.60% random coil for XBB.1.5; 5.48% α-helix, 27.40% β-sheet, and 67.12% random coil for BA.5; and 6.85% α-helix, 20.55% β-sheet, and 72.60% random coil for B.1.617.2, respectively. These secondary structural variations account for the observed differences in overall conformation and the ACE2-binding interface compared with individual monomeric RBDs or homotrimers.

For protein expression, Expi293 cells were transfected with pAd/TriRBD plasmid. Five days post-transfection, the cell supernatants were harvested, and the recombinant TriRBD was purified using a C-tagXL affinity matrix. Successful purification was confirmed by silver staining (Figure 1C). TriRBD was recognized by a monoclonal anti-Ctag antibody, appearing as a diffuse band with a molecular weight of approximately 100 kDa under denaturing and reducing conditions. This migration pattern suggested that the protein exists as a glycosylated monomeric form (Figure 1D). Next, MAPs loaded with 5 μg of TriRBD (MAP-TriRBD) were fabricated using a hyaluronic acid-based droplet extension (DEN) technique. A subset these MAPs underwent sterilization by gamma irradiation. The DEN-fabricated MAP features a double-layered structure, consisting of a base layer (900 μm in width and 200 ± 20 μm in height) and an upper layer (600 ± 20 μm in length). Each 2.0 cm^2^ patch contained a 5 × 5 arrays covering an effective area of 1.76 cm^2^, with an average needle length of 825 ± 25 μm. The mechanical strength (fracture force) of the MAP was measured to exceed 0.1 N, conforming sufficient structural integrity to penetrate the skin (Figure 1E).

### 3.2. Immunogenicity of MAP-TriRBD in BALB/c Mice

**Figure 2:**
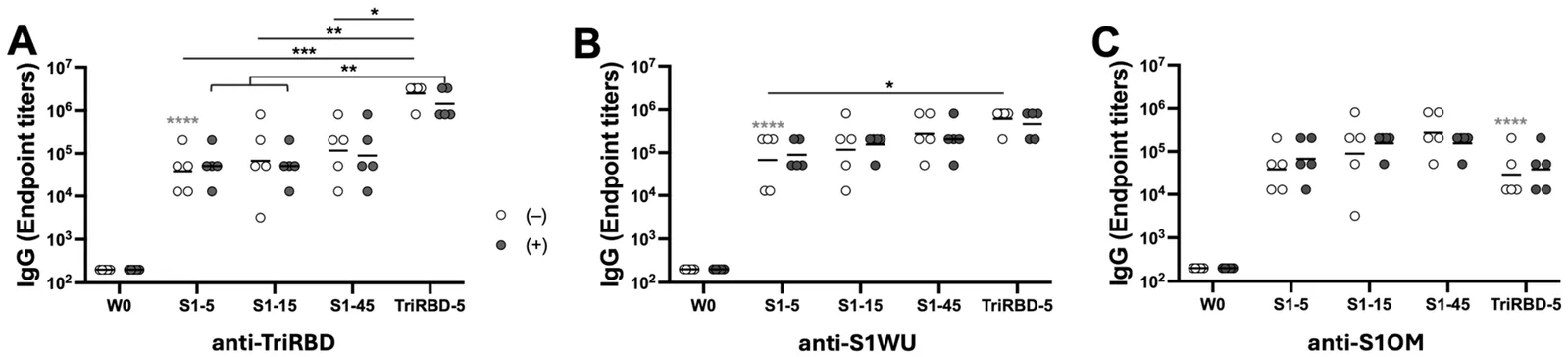
Comparison of Immune responses induced by different dose of MAP-rS1RS09Delta and MAP-TriRBD. Balb/c mice (5 per group) were prime and boosted three weeks intervals with either non-irradiated (−, blank circle) or irradiated (+, grey circle) of 5, 15, 45 μg of MAP-rS1RS09Delta (S1–5, S1–15, or S1–45, respectively) and 5 μg of MAP-TriRBD. TriRBD (~100 kDa) contains three RBD copies per molecule, whereas S1RS09Delta (~110 kDa) has a single RBD copy per molecule. Thus, comparing 5 µg of TriRBD to 15 µg of MAP-S1RS09Delta provides a near-identical molar equivalent dose of the functional target antigen (RBD epitopes). Reciprocal serum endpoint dilutions of TriRBD-specific (**A**), SARS-CoV-2 S1WU-specific (**B**), and SARS-CoV-2 S1OM-specific (**C**) antibodies were measured by ELISA at week 16 post-prime immunization. Individual data points represent each mouse. Antibody endpoint titers were log-transformed prior to statistical analysis, and the bars denote geometric mean. Black asterisks indicate significant differences between groups analyzed by one-way ANOVA with Tukey’s post-test (* *p* < 0.05, ** *p* < 0.01, *** *p* < 0.001). Grey asterisks represent statistical differences compared to pre-immunized sera (**** *p* < 0.0001).

To assess the immunogenicity of MAP-TriRBD, BALB/c mice (n = 5 per group) were immunized via a prime-boost regimen at three-week interval using either non-irradiated (−) or irradiated (+) MAPs containing 5 μg of TriRBD. Sera were collected prior to immunization (week 0) and at week 16 post-prime, and TriRBD-specific IgG end pointe titers (EPT) were determined by ELISA. For comparative analysis, serum samples from mice immunized with 5 μg, 15 μg, or 45 μg of MAP-S1RS09Delta [9] were also evaluated. At week 16, all vaccinated groups exhibited significantly higher TriRBD-specific IgG EPT compared to week 0 (*p* < 0.0001). Notably, MAP-TriRBD elicited significantly higher EPT than most MAP-S1RS09Delta groups. In contrast, the MAP-S1RS09Delta groups achieved the similar, lower geometric mean titers (GMT) regardless of the dose. No significant differences were observed between non-irradiated and irradiated MAPs in any group (Figure 2A).

Next, we evaluated the IgG EPT specific to Wuhan-Hu-1 S1 (S1WU) (Figure 2B) or Omicron BA.1 S1 (S1OM) antigen (Figure 2C). Against Wuhan S1, all vaccinated groups showed a significant increase in IgG EPT at week 16 compared to week 0 (*p* < 0.0001). Interestingly, MAP-TriRBD achieved a superior GMT compared to the 15 μg (a near-identical molar equivalent dose to 5 μg of TriRBD regarding the functional target, RBD epitopes) and 45 μg (three-fold molar excess) MAP-S1RS09Delta groups, although the differences between these groups did not reach statistical significance. As observed previously, gamma irradiation had no impact on the antibody response.

Against the Omicron S1 antigen, all vaccinated groups showed significantly elevated IgG EPT at week 16 compared to week 0 (*p* < 0.0001). However, S1OM-specific IgG EPT of MAP-TriRBD group was the lowest among the groups, appearing comparable to the 5 μg MAP-S1RS09Delta group. No significant differences were observed between the groups for this specific antigen. These results suggest that TriRBD delivered via MAP induced a superior antibody response against the parental SARS-CoV-2 S1WU, despite using a lower vaccine dose. Conversely, the lower immunogenicity observed against Omicron S1 highlights the necessity for future adjuvant application to broaden the spectrum of the immune response.

### 3.3. Preparation of Adjuvanted MAPs

**Figure 3:**
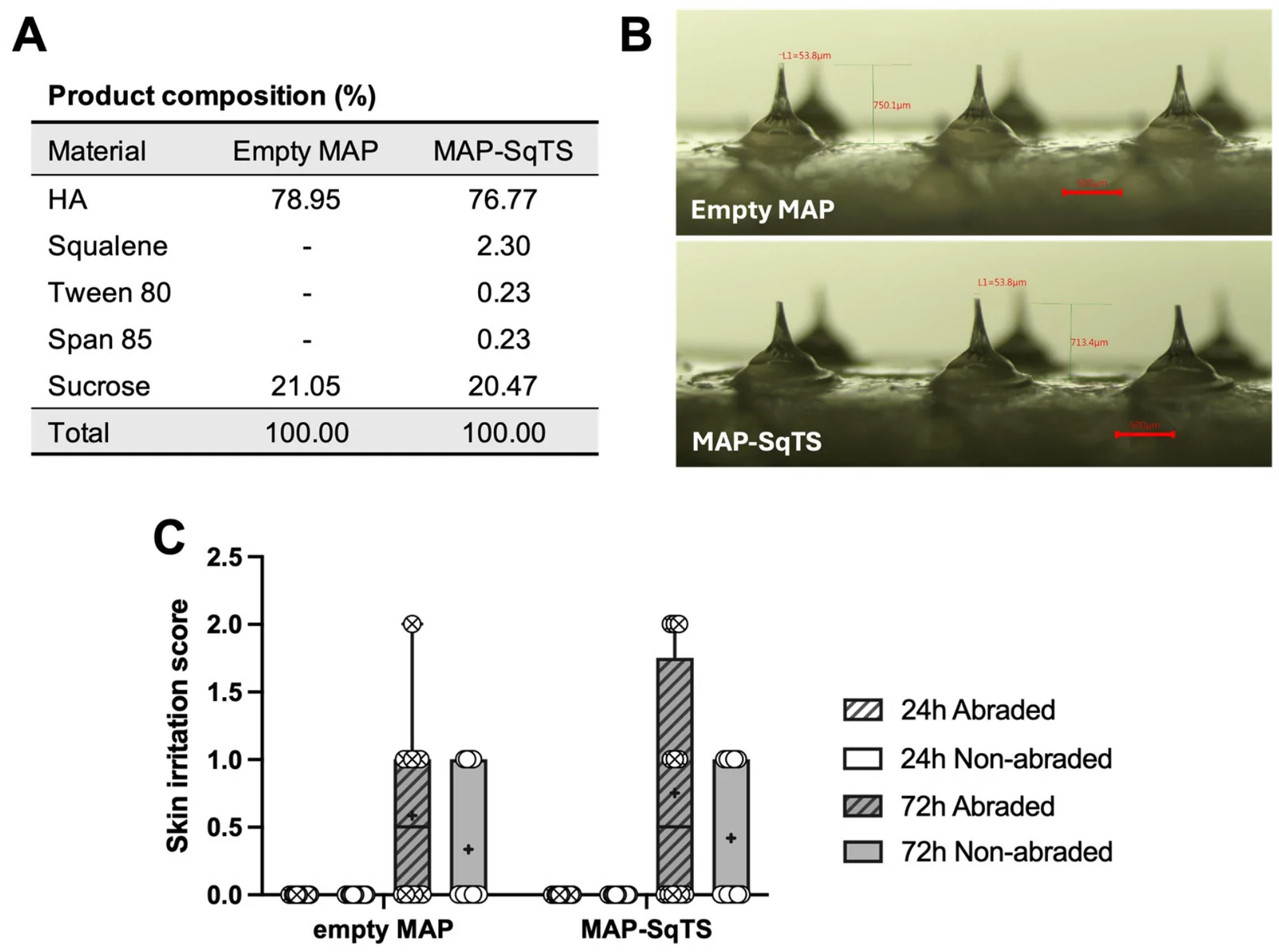
Assessment of skin irritation induced by SqTS-loaded MAPs. (**A**) Composition of microneedle patch formation of empty MAP and MAP-SqTS (**B**) Each MAP was occupied in a 1.76 cm^2^ area size of 5 × 5 arrays of 710–750 μm-long microneedles with and without SqTS emulsion adjuvant in circular patterned film (2.0 cm^2^) (**C**) Skin erythema and edema were evaluated on albino rabbit at 24 h and 72 h post-administration using the Draize scoring system: 0, no erythema/edema; 1, very slight erythema/edema; 2, well-defined erythema/edema; 3, moderate to severe erythema/edema and 4, sever erythema/edema. Small crosses denote mean values (n = 6).

To enhance the immunogenicity of the MAP formulation, an oil-in-water (O/W) emulsion was selected as the adjuvant. This choice was informed by the clinical approval of MF59-adjuvanted influenza vaccine for elderly patients and young child [35,36]. To evaluate the feasibility of incorporating an adjuvant into the MAP structure, we first formulated a squalene-based O/W emulsion (SqTS). The SqTS formulation consisted of 5.0% squalene (emulsifier), 0.5% Tween-80, and 0.5% Span-85 (non-ionic surfactants) in a 10 mM Citric buffer system (pH 6.5) (Figure 3A). The mixture was processed using high-pressure homogenization to achieve a stable 5% (*v*/*v*) emulsion. Subsequently, we manufactured both SqTS-loaded and empty (control) MAPs using the droplet extension technique (DEN) as previously described [8,9]. The dissolving MAPs featured a circular design with 1.6 cm diameter (total patch area: 2.0 cm^2^). Each MAP contained a 5 × 5 needles array covering an active area of 1.76 cm^2^, with an overall needle length of approximately 750 ± 50 μm (Figure 3B). Mechanical integrity was evaluated using fracture force measurements. The forces exceeded 0.1 N, which is the minimum threshold required for effective skin penetration, confirming the structural suitability of the adjuvanted MAPs.

### 3.4. Skin Irritation Study of Adjuvanted MAPs

To evaluate the potential for skin irritation following transdermal administration of SqTS-adjuvanted MAP, an in vivo study was conducted using six albino rabbits. Skin irritation was assessed using the Draize Scoring method, with the rabbit skin rendered hairless prior to patch application. Observations for erythema and edema were recorded at 24 and 72 h post-application, with primary skin irritation (PSI) scores assigned on a scale of 0 to 4. After 24 h, no skin irritation was detected in areas treated with either emptyMAP or MAP-SqTS. At 72 h, the PSI scores for emptyMAP were 0.58 ± 0.67 for the abraded sites and 0.33 ± 0.49 for the non-abraded sites. For MAP-SqTS, the scores were 0.75 ± 0.87 (abraded) and 0.42 ± 0.51 (non-abraded) (Figure 3C). Notably, all mean scores remained below 1.5, the threshold for “No classification (mean scores below 1.5 of for erythema or for edema in at least 2 of 3 tested animals)” under the United Nations Globally Harmonized System of Classification and Labeling of Chemicals (UN GHS) for skin irritation hazard. These results indicated that microneedle patches incorporating the SqTS adjuvant are considered non-irritating [37]. The macroscopic appearances of the treated area are presented in Supplementary Figure S1.

The Primary irritation index (PII) values are summarized in Table 2. The PII for empty MAP was 0.46, classifying it as a non-irritant (primary dermal irritation index [PDII] range of 0 to 0.5). In contrast, the MAP-SqTS exhibited a PII of 0.58, classifying it as a slight irritant (PDII range of >0.5 to 2.0). Although the MAP-SqTS score falls within the “slight irritation” range, it remains near the borderline of the non-irritation category and shows no significant difference compared to the empty MAP control. Collectively, these findings suggested that the SqTS-adjuvant MAP formulation is non-toxic, non-irritant, and biocompatible.

### 3.5. Immunogenicity of Adjuvanted MAP-TriRBD

Building on the safety profile of MAP containing oil-in-water emulsion, we extended our application to include two specific adjuvant formulations, as follows: SqTS (5.0% squalene, 0.5% Tween-80, 0.5% Span-85) and SqT (5.0% squalene, 1.0% Tween-80) (Supplementary Figure S2A). The average droplet diameters of the SqTS and SqT emulsions were 172.7 nm and 159.5 nm, respectively, showing high similarity to commercial adjuvants such as AddaVax (Invitrogen, 160.0 nm) and MF59 (Supplementary Figure S2B) [38]. Both emulsions exhibited a mean polydispersity index (PdI) of less than 0.1, indicating a highly homogeneous size distribution, although SqT demonstrated slightly less uniformity compared to SqTS (Supplementary Figure S2C).

**Figure 4:**
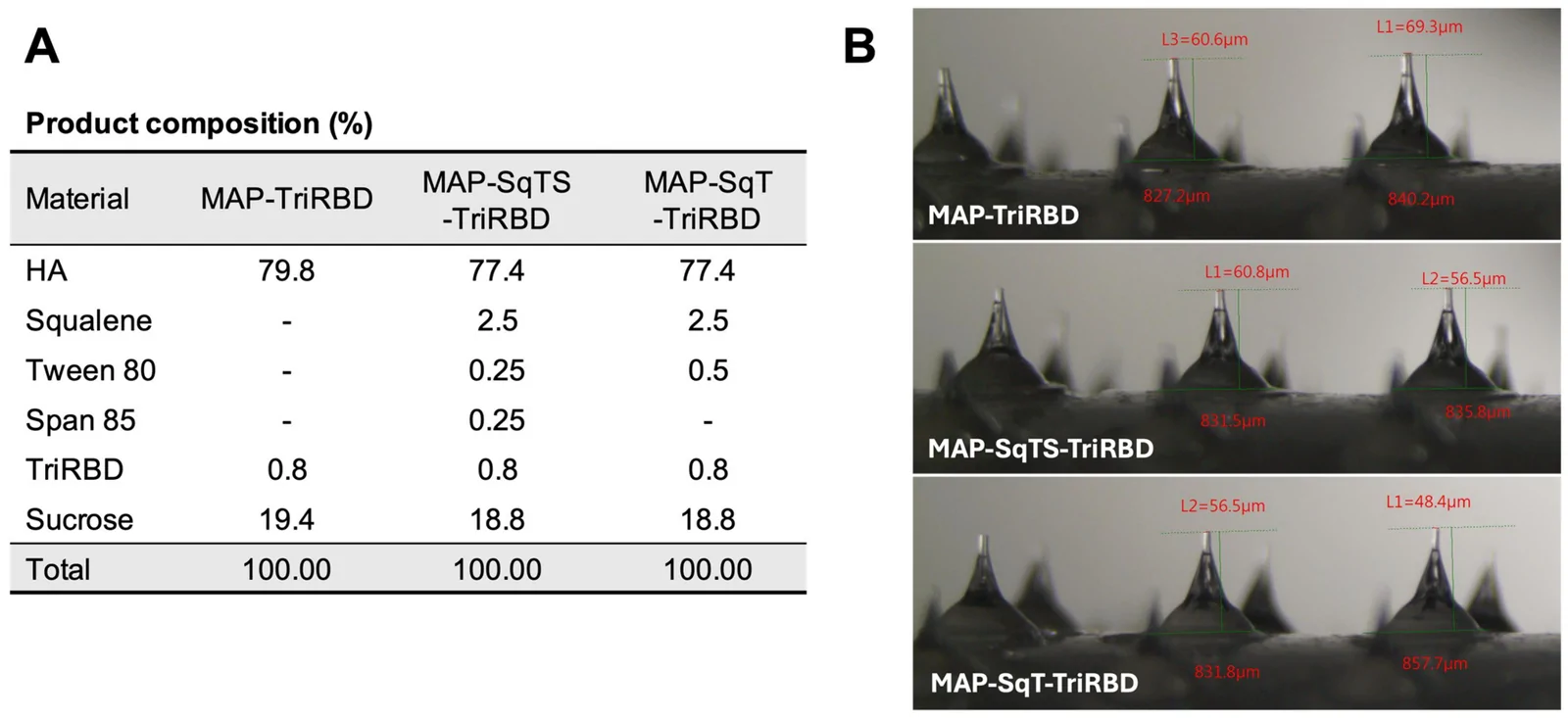
Fabrication of dissolving microneedle array patch of TriRBD with/without SqT or SqTS emulsion adjuvants (**A**) composition of MAP-TriRBD, MAP-SqT-TriRBD, and MAP-SqTS-TriRBD (**B**) Image of MAPs. Each MAP was approximately 830 μm in length, occupying a 5 × 5 array of 1.76 cm^2^ area in circular patterned film (2.0 cm^2^).

Subsequently, MAPs loaded with either SqTS or SqT and 5 μg of TriRBD (MAP-SqTS-TriRBD and MAP-SqT-TriRBD) were fabricated. The final composition featured a 1:1 mixture of TriRBD and the respective adjuvant (Figure 4A). These circular MAPs (1.6 cm diameter, 2.0 cm^2^ total area) consisted of a 5 × 5 microneedle arrays with an overall length of 820~850 μm. The double layered structure comprised a base (900 μm in width, 200 ± 20 μm in height) and an upper layer (600 ± 20 μm in length), covering an active area of 1.76 cm^2^ (Figure 4B). The fracture force of the needles exceeded 0.1 N, confirming that the incorporation of either SqTS or SqT did not compromise the mechanical strength required for skin penetration.

**Figure 5:**
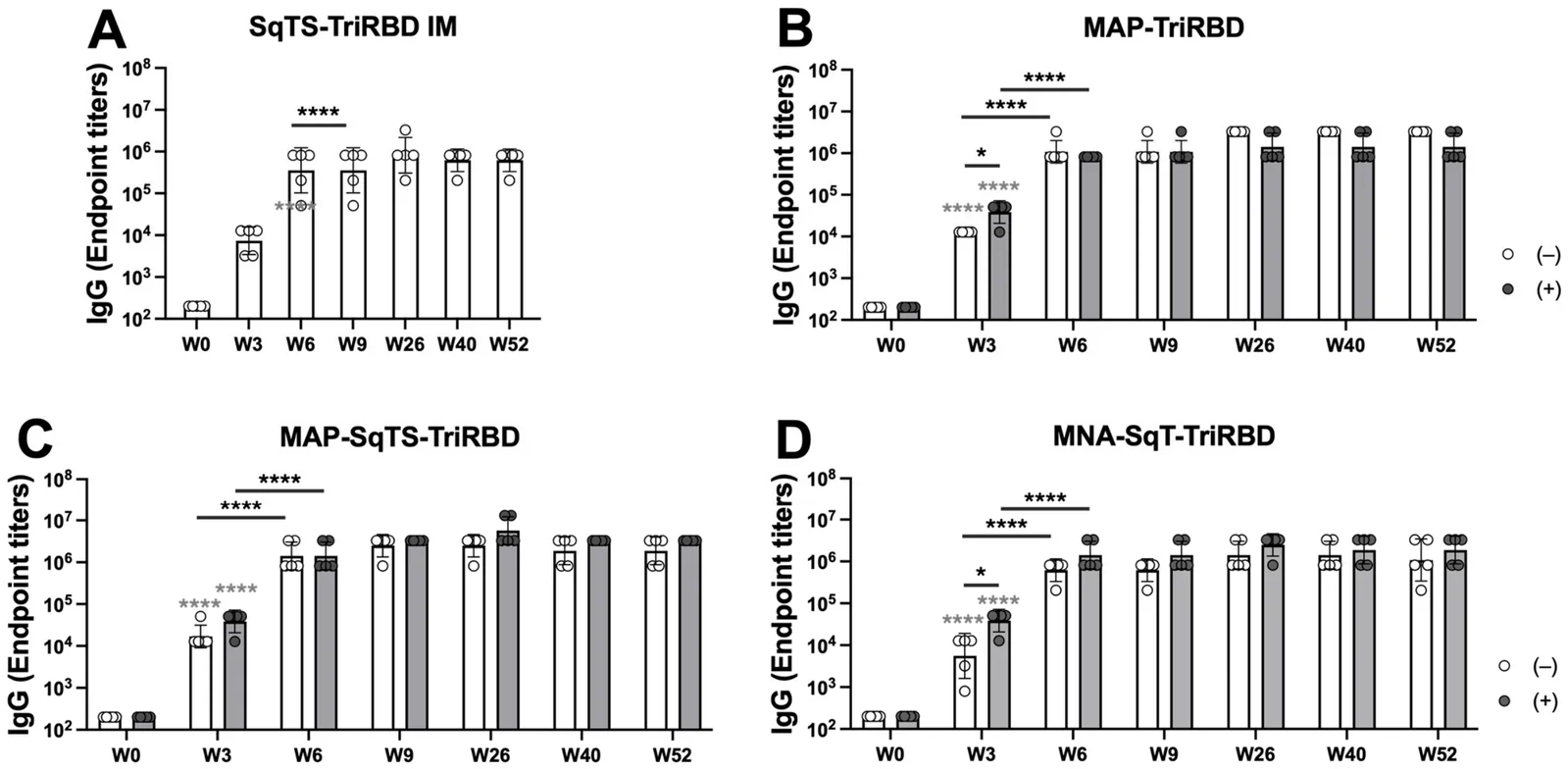
Comparison of humoral immune response induced by different route and presence of adjuvants. Balb/c mice (5 per group) were prime and boosted three weeks intervals with 5 μg of TriRBD and SqTS intramuscularly (**A**) and with either non-irradiated (−, blank circle) or irradiated (+, grey circle) of MAP-TriRBD (**B**), MAP-SqTS-TriRBD (**C**), and MAP-SqT-TriRBD (**D**). Reciprocal serum dilutions of TriRBD-specific antibodies were measured by ELISA to determine the IgG endpoint titers at weeks 0, 3, 6, 9, 26, 40, and 52. The titers at each time points were showed for each mouse with dots. Antibody endpoint titers were log-transformed prior to statistical analysis. The graph represents geometric mean ± geometric SD. Groups were compared by one-way ANOVA with Tukey’s post-test (* *p* < 0.05, **** *p* < 0.0001). Grey asterisks represent statistical differences compared to pre-immunized sera (**** *p* < 0.0001).

To evaluate long-term immunogenicity, BALB/c mice (n = 5 per group) were immunized via a prime-boost regimen at a three-week interval. Groups received either non-irradiated (−) or irradiated (+) of MAP-TriRBD, MAP-SqTS-TriRBD, and MAP-SqT-TriRBD via the dorsal skin. For comparison, one group was immunized intramuscularly (IM) with 5 μg of TriRBD mixed 1:1 with SqTS adjuvant. Sera were collected at baseline (week 0) and at weeks 3, 6, 9, 26, 40, and 52 post-prime (Figure 5). All vaccinated groups showed significantly elevated TriRBD-specific IgG EPT after both prime and boost immunization (*p* < 0.0001). The geometric mean titers (GMTs) peaked at week 26 and remained significantly higher than baseline at week 52, indicating a robust and sustained immune response. Notably, all MAP-delivered groups regardless of adjuvant exhibited higher GMTs compared to the IM injection group. While no significant differences were generally observed between non-irradiated and irradiated MAPs, irradiated MAP-SqT-TriRBD and MAP-TriRBD groups showed significantly higher GMTs at week 3. Interestingly, for the adjuvanted groups, irradiated MAPs tended to elicit higher GMTs throughout most of the study period, whereas the opposite trend was observed in non-adjuvanted MAP-TriRBD group.

### 3.6. IgG Subtype Profiles and Th2/Th1 Immune Responses

**Figure 6:**
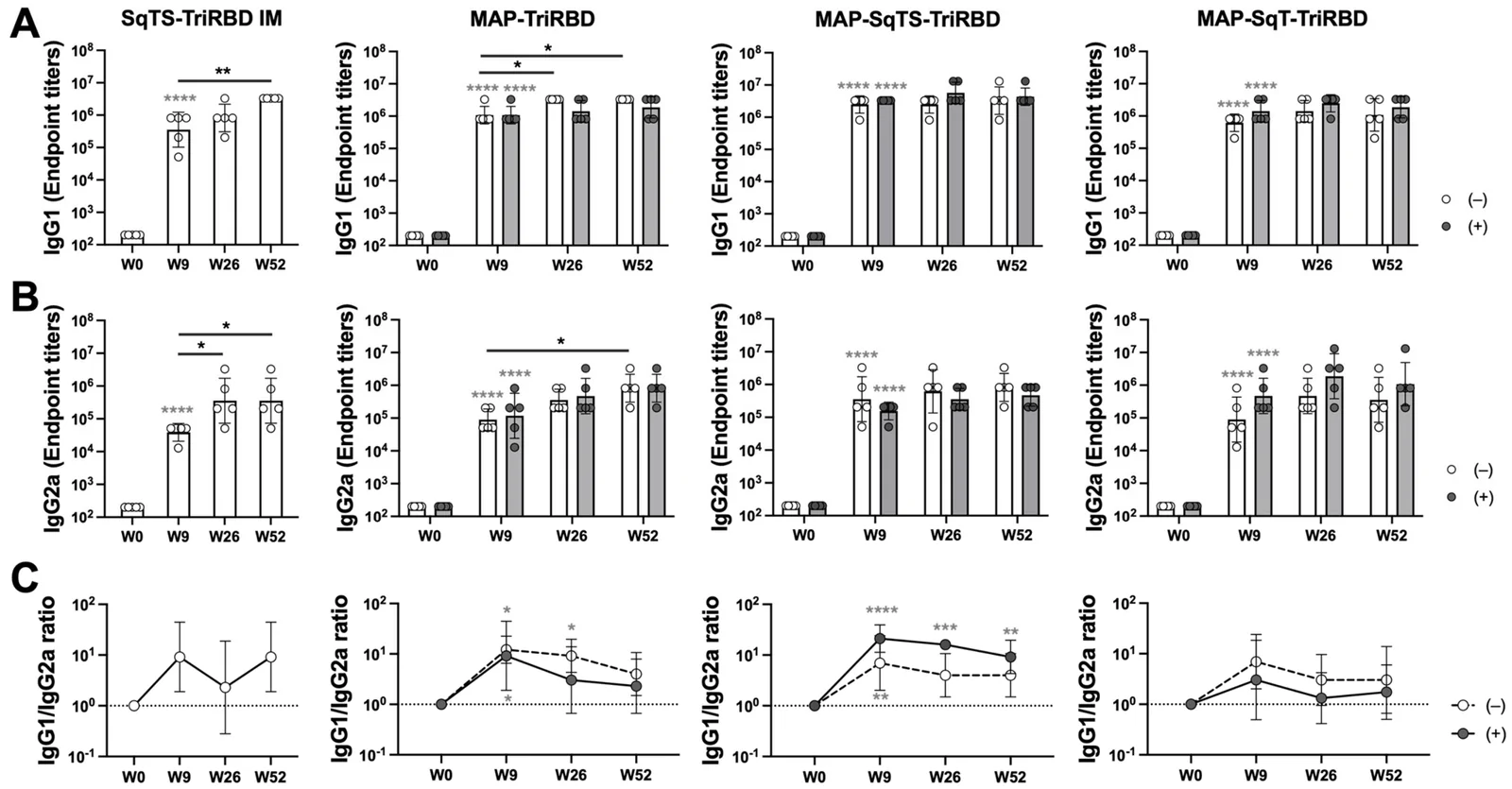
TriRBD-specific IgG subclasses. Sera were collected from the mice immunized with 5 μg of TriRBD and SqTS intramuscularly (SqTS-TriRBD IM) and with either non-irradiated (−, blank circle) or irradiated (+, grey circle) of MAP-TriRBD, MAP-SqTS-TriRBD, and MAP-SqT-TriRBD at weeks 0, 9, 26, and 52. TriRBD-specific IgG1 (**A**) and IgG2a (**B**) were assessed by ELISA to determine each IgG subclasses’ endpoint titer. The titers at each time points were showed for each mouse. The graph represents geometric mean ± geometric SD. (**C**) TriRBD-specific IgG1/IgG2a ratios of individual mice at each of the time points as geometric mean ± geometric SD. The dotted horizontal line indicates an IgG1/IgG2a ratio of 1. Significant differences among groups were evaluated by one-way ANOVA with Tukey’s post-test and indicated by * *p* < 0.05 and ** *p* < 0.01. Grey asterisks represent statistical differences compared to pre-immunized sera (* *p* < 0.05, ** *p* < 0.01, *** *p* < 0.001, **** *p* < 0.0001).

We further analyzed TriRBD-specific IgG1 and IgG2a titers to determine whether the use of adjuvants or gamma irradiation influenced the balance Th2- or Th1-like immune response (Figure 6). Sera collected at weeks 0, 9, 26, and 52 post-prime immunization were subjected to isotype-specific ELISA. All vaccinated groups exhibited significantly higher TriRBD-specific IgG1 GMT at all time points compared to pre-immune sera. Notably, only non-irradiated (−) MAP-TriRBD and the IM (TriRBD + SqTS) groups showed a significant increase IgG1 GMT at week 52 compared to week 9, whereas titers in all other groups remained consistently high throughout the entire observation period (Figure 6A). Similar patterns also were observed for the IgG2a subclass (Figure 6B).

The TriRBD-specific IgG1/IgG2a ratios were then calculated to assess the immune polarization (Figure 6C). Both non-irradiated (−) and irradiated (+) of MAP-TriRBD groups exhibited a predominantly Th2-biased responses at week 9, which shifted toward a more balanced Th2- or Th1-like profile at later time points. A similar trend was observed in non-irradiated (−) MAP-SqTS-TriRBD group. In contrast, the irradiated (+) MAP-SqTS-TriRBD group maintained a significantly Th2-prevalent response across all time points. Interestingly, both non-irradiated (−) and irradiated (+) MAP-SqT-TriRBD groups demonstrated a consistently balanced immune response, comparable to the IM group. These findings indicate that irradiated MAP-SqT-TriRBD elicits the most balanced immune response based on the IgG1/IgG2a ratio, suggesting that SqT is an ideal adjuvant for protein subunit vaccines delivered via irradiated dissolving microneedle patch.

### 3.7. Omicron-S1-Specific IgG Levels to Adjuvanted MAP-TriRBD

**Figure 7:**
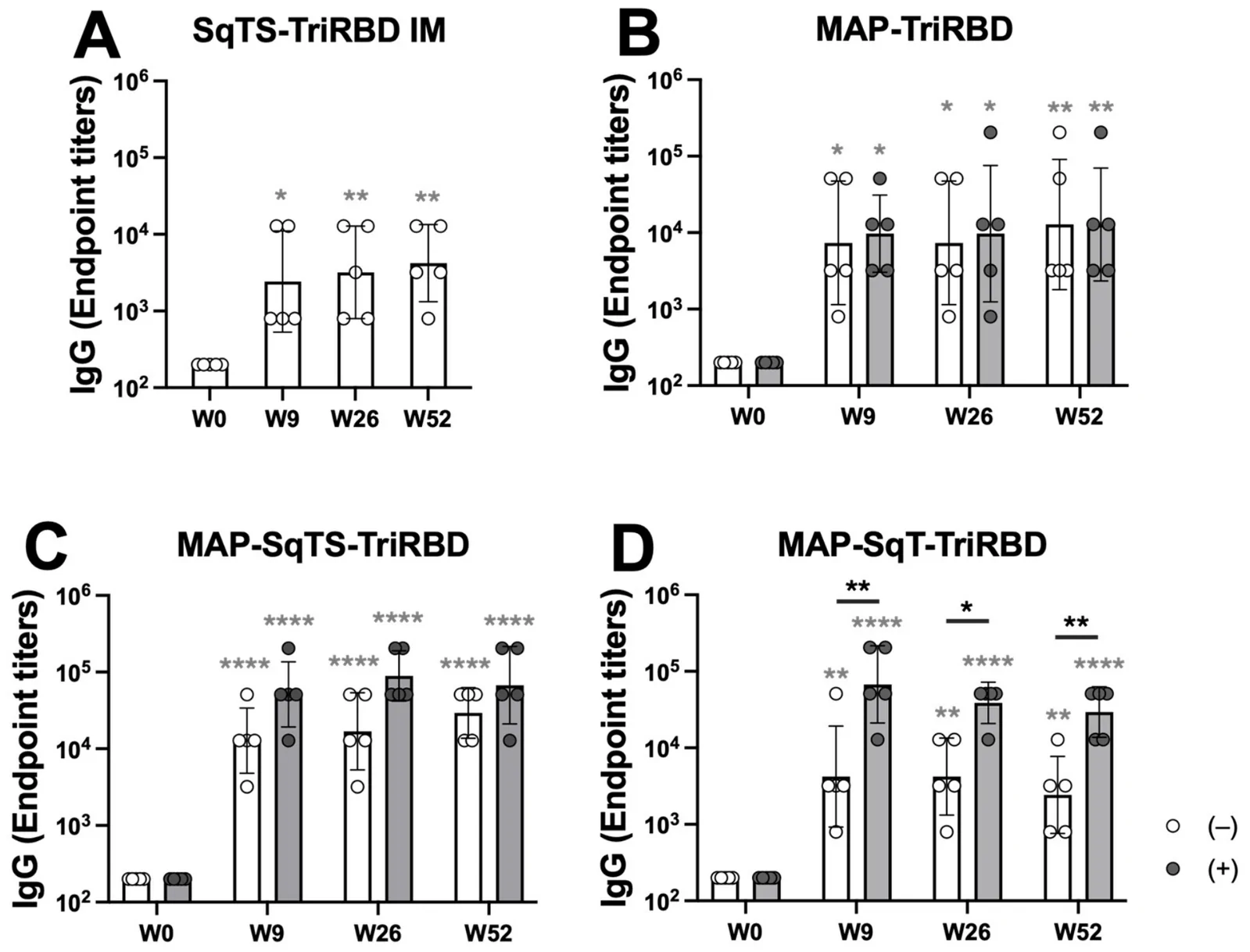
Comparison of S1OM-specific humoral immune response induced by different route and presence of adjuvants. Balb/c mice (5 per group) were prime and boosted three weeks intervals with 5 µg of TriRBD and SqTS intramuscularly (**A**) and with either non-irradiated (−, blank circle) or irradiated (+, grey circle) of MAP-TriRBD (**B**), MAP-SqTS-TriRBD (**C**), and MAP-SqT-TriRBD (**D**). Reciprocal serum endpoint dilutions of SARS-CoV-2 S1OM-specific antibodies were measured by ELISA to determine the IgG endpoint titers at weeks 0, 9, 26, and 52. The titers at each time points were showed for each mouse with dots. The graph represents geometric mean ± geometric SD. Groups were compared by one-way ANOVA with Tukey’s post-test. Significant differences are indicated by * *p* < 0.05 and ** *p* < 0.01. Grey asterisks represent statistical differences compared with pre-immunized sera (* *p* < 0.05, ** *p* < 0.01, **** *p* < 0.0001).

We determined the IgG antibody endpoint titers (EPT) specific to the Omicron BA.1 S1 (S1OM) protein in the sera of vaccinated mice (Figure 7). Sera collected at baseline (weeks 0) and at weeks 9, 26, and 52 post-prime immunization were analyzed using S1OM-specific ELISA. All vaccinated groups exhibited significantly higher S1OM-specific IgG EPT at all examined time points compared to week 0. MAP-TriRBD (both ± irradiation) and the IM (TriRBD + SqTS) groups showed a more pronounced increase in IgG GMT at week 52 (*p* < 0.01) than week 9 (*p* < 0.05). Notably, Adjuvanted MAP-SqTS-TriRBD (both ± irradiation) and irradiated MAP-SqT-TriRBD groups showed significantly elevated S1OM-specific IgG EPT throughout the study (*p* < 0.0001, vs. week 0). Interestingly, the irradiated (+) MAP-SqT-TriRBD group achieved superior GMTs compared to non-irradiated (−) counterpart (*p* < 0.01), indicating a significant impact of irradiation on the specific formulation. In contrast, no significant differences were observed between non-irradiated and irradiated versions in the MAP-TriRBD and MAP-SqTS-TriRBD groups.

**Figure 8:**
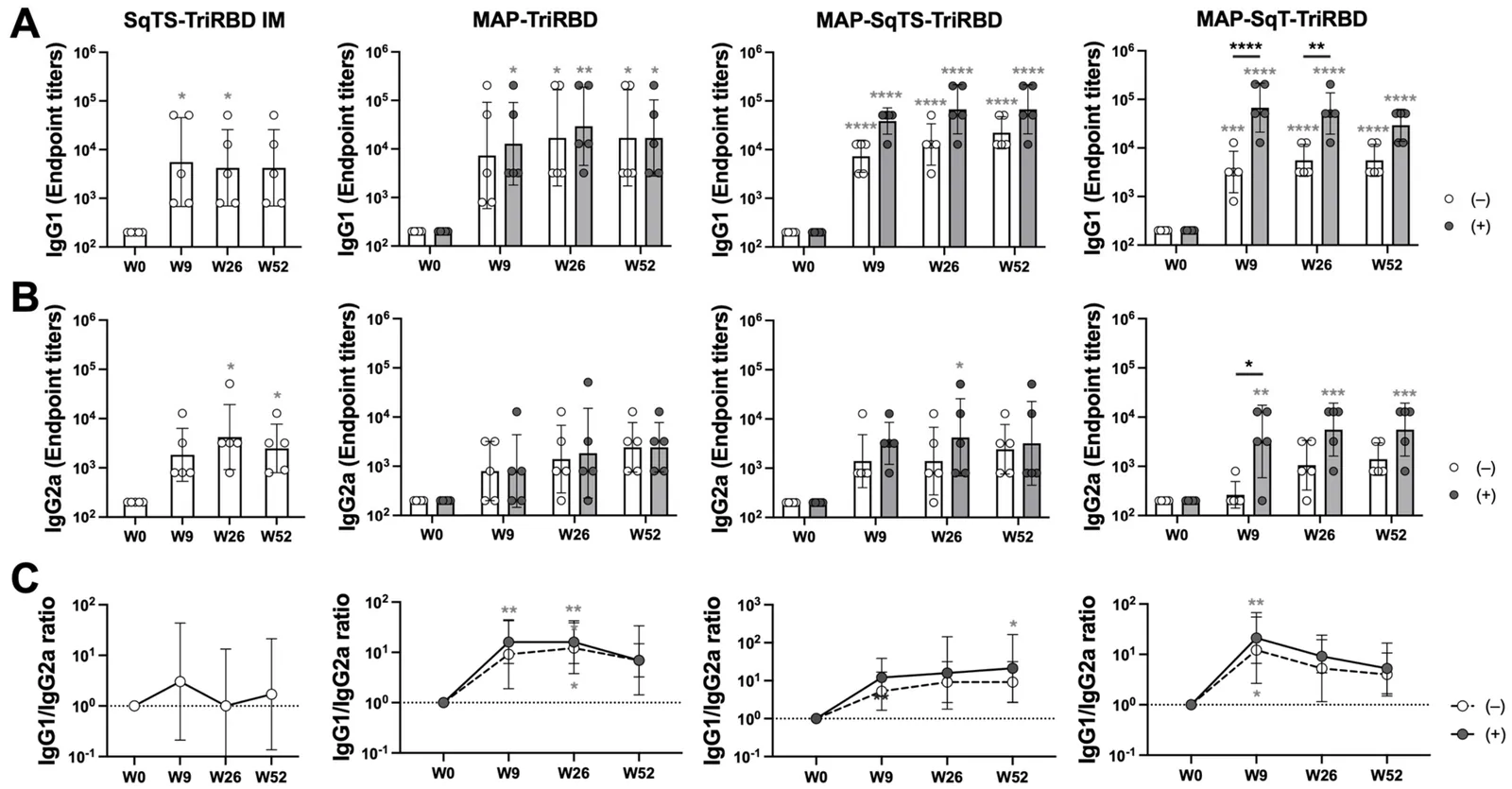
SARS-CoV-2 S1OM-specific IgG subclasses. Sera were collected from the mice immunized with 5 μg of TriRBD and SqTS intramuscularly (SqTS-TriRBD IM) and with either non-irradiated (−, blank circle) or irradiated (+, grey circle) of MAP-TriRBD, MAP-SqTS-TriRBD, and MAP-SqT-TriRBD at weeks 0, 9, 26, and 52. S1OM-specific IgG1 (**A**) and IgG2a (**B**) were assessed by ELISA to determine each IgG subclasses’ endpoint titer. The titers at each time points were showed for each mouse. The graph represents geometric mean ± geometric SD. (**C**) S1OM-specific IgG1/IgG2a ratios of individual mice at each of the time points as geometric mean ± geometric SD. The dotted horizontal line indicates an IgG1/IgG2a ratio of 1. Significant differences among groups were evaluated by one-way ANOVA with Tukey’s post-test and are indicated by * *p* < 0.05, ** *p* < 0.01, and **** *p* < 0.0001. Grey asterisks represent statistical differences compared to pre-immunized sera (* *p* < 0.05, ** *p* < 0.01, *** *p* < 0.001, **** *p* < 0.0001).

We further examined the S1OM-specific IgG1 and IgG2a titers to assess the influence of adjuvants and gamma irradiation on antibody isotypes (Figure 8). All vaccinated groups, except for the IM group at week 52 and the non-irradiated MAP-TriRBD group at week 9, showed significantly higher S1OM-specific IgG1 GMTs at all time points compared to pre-immune sera (Figure 8A). Specially, the adjuvanted MAP-SqTS-TriRBD and MAP-SqT-TriRBD groups exhibited highly significant increase (*p* < 0.0001), while the MAP-TriRBD and IM groups showed more moderate elevations (*p* < 0.05). Notably, the irradiated MAP-SqT-TriRBD group elicited significantly higher GMTs than the non-irradiated group at weeks 9 and 26 (*p* < 0.0001 and *p* < 0.01, respectively). Regarding the IgG2a subclass, only the irradiated (+) MAP-SqT-TriRBD showed a superior increase in S1OM-specific IgG2a GMT throughout the entire observation period and achieved significantly more increase GMTs compared to non-irradiated (−) counterpart at week 9 (*p* < 0.05). IM groups at weeks 26 and 52 and the irradiated MAP-SqTS-TriRBD group at week 26 showed significantly moderate increases (*p* < 0.05) compared at week 0, whereas other groups failed to induce a significant IgG2a response across all time points (Figure 8B).

The S1OM-specific IgG1/IgG2a ratios indicated that the IM group showed balanced Th2- or Th1-like immune response all time points, although with a wide variability. Both the MAP-SqT-TriRBD and MAP-TriRBD groups initially showed a predominantly Th2-biased response but shifted toward a balanced profile by weeks 26 and 52, respectively. In the MAP-SqTS-TriRBD groups, the non-irradiated (−) version maintained a balanced response throughout and the irradiated (+) version was balanced at weeks 9 and 26 before shifting toward to Th2-prevalent response by week 52 (*p* < 0.05) (Figure 8C). These results demonstrate that TriRBD delivered via MAP with the SqT adjuvant induced high levels of S1OM-specific IgG1 and IgG2a, resulting in a balanced immune response against the antigenically distinct SARS-CoV-2 Omicron S1. Collectively, these findings reinforce SqT as an ideal adjuvant for protein subunit vaccines delivered via irradiated dissolving microneedle array patches.

## 4. Discussion

The development of an effective multivalent vaccine platform is critical for addressing the rapid antigen evolution of SARS-CoV-2. In this study, we demonstrated that a microneedle array patch (MAP) loaded with a trivalent recombinant protein (TriRBD) and a novel oil-in-water adjuvant (SqT) induces robust, balanced, and long-lasting immune responses.

Our results indicated that the TriRBD construct—comprising XBB.1.5, BA.5, and Delta RBDs—elicited superior antibody titers compared to the previous S1-based monovalent constructs. This suggests that the sequential arrangement of diverse RBDs separated by flexible (GGGGS)_3_ linkers maintains the structural integrity of individual epitopes while increasing the molar efficiency of the antigen; notably, MAP-TriRBD induced significantly higher IgG EPT than the MAP-S1RS09Delta group despite using a lower overall dose against ancestral S1WU (Figure 2). This potency can be attributed to the dense concentration of critical neutralizing epitopes within the trivalent design. Thereby, multivalent vaccines with epitope diversity may theoretically enhance the magnitude and breath of cross-neutralization responses, providing a buffer for unpredictable future variants. However, the relatively low IgG EPT observed against the Omicron BA.1 S1 antigen in the MAP-TriRBD group can be attributed to several immunological factors. First, the antigenic distance between the vaccine components (Delta, BA.5, and XBB.1.5) and the BA.1 subvariant may limit cross-reactive binding [39,40]. Second, since TriRBD is an RBD-focused immunogen, it lacks the N-terminal domain (NTD) and C-terminal domain (CTD) sequences present in the full S1 protein used for ELISA coating. Consequently, this epitope-focused approach successfully targets the critical receptor-binding site. However, it shows lower total antibody capture in assays involving the full S1 domain compared to vaccines using the entire S1 subunit [41,42,43].

MF59 were evaluated as a safe and effective adjuvant in intramuscular injection, leading to approval by FDA [22,44]. An adjuvant safety with different administration route is important. Here, the safety profile of the intradermal inoculation via microneedle array patch (MAP-SqTS) formulation was confirmed through Draize scoring in rabbits, showing no significant irritation (PII < 0.6) and full biocompatibility (Figure 3). Furthermore, the maintenance of mechanical strength (>0.1 N) and immunogenicity following gamma irradiation addresses a major hurdle in MAP commercialization, namely terminal sterilization. The stability of the protein-adjuvant complex under these conditions suggests that this platform is suitable for mass production and global distribution without the need for a stringent cold chain.

A key finding of this study is that MAP-TriRBD without adjuvant outperformed traditional intramuscular injection with adjuvant in terms of IgG geometric mean titers (Figure 4). The skin is highly enriched with professional antigen-presenting cells (APCs), such as Langerhans cells and dermal dendritic cells. The hyaluronic-acid-based DEN technique allows for the precise deposition of TriRBD into these immune-rich layers, facilitating efficient antigen uptake and migration to draining lymph nodes. The sustained antibody levels observed up to week 52 post-prime further highlight the ability of MAPs to establish long-term immune memory, a significant advantage over the waning immunity often associated with conventional liquid injectables. These results are in line with our previous studies [9], although intramuscular injection was performed without adjuvant.

We assessed the immunogenicity of TriRBD delivered via both irradiated and non-irradiated MAPs formulated with two types of squalene-based O/W emulsions (SqTS or SqT). Squalene is naturally synthesized in the pathway of human steroid hormones, and is present in skin, adipose tissue, and muscles [45,46]. The natural squalene is obtained from shark liver and prepared for vaccine adjuvants after purification [47]. Additionally, the plant-derived pharmaceutical surfactants Tween 80 and Span 85 (Sorbitan Trioleate) combine with squalene to form safe, biodegradable, and well-tolerated formulations. Both surfactants are responsible for MF59-induced transient ATP release at intramuscular injection site, which is a crucial mechanism of action that promotes efficient CD4 T-cell priming and high antibody titers [48]. However, the surfactant mixture alone (0.5% Span 85 and 0.5% Tween 80 in PBS) fails to induce a comparable adjuvant effect when co-administered with trivalent influenza vaccine [49]. Span 85 is a non-ionic, lipophilic (oil-soluble) surfactant, stabilizing dispersions when combined with hydrophilic (water-soluble) surfactant like Tween 80 due to optimal HLB (Hydrophilic-Lipophilic Balance). However, squalene-based emulsion adjuvant in MAP is less important to keep the structural stability as in the liquid buffer. This is the reason we exclude Span 85 from the SqTS.

Regarding irradiation, MAP-TriRBD without adjuvant showed a similar immune response against TriRBD in both non-irradiated and irradiated groups, even slightly higher in the non-irradiated group. However, regarding adjuvated MAP-SqTS-TriRBD and MAP-SqT-TriRBD groups, a slightly higher geometric mean titer was determined in irradiated groups, although no significant differences were observed (Figure 5). These patterns were observed more clearly in ELISA against S1OM (Figure 7). Interestingly, MAP-SqT-TriRBD (+) group showed statistically significant higher GMT compared to MAP-SqT-TriRBD (−) (Figure 7D). Furthermore, MAP-SqTS-TriRBD (+) showed significant Th2 prevalent immune response among the tested animal groups (Figure 6 and Figure 8), while MAP-SqT-TriRBD (+) group exhibited the most balanced Th1/Th2 immune response, characterized by elevated levels of both IgG1 and IgG2a. This balance is vital for viral clearance and the prevention of vaccine-associated enhanced respiratory disease (VAERD). Our data suggest that SqT is particularly durable to gamma irradiation, as the irradiated (+) MAP-SqT-TriRBD group achieved superior GMTs against the antigenically distant Omicron S1 compared to non-irradiated versions. The enhanced immunogenicity of the irradiated (+) MAP-SqT-TriRBD group suggests that SqT may facilitate the formation of stable antigen-adjuvant complexes within the solid hyaluronic acid matrix. Unlike aqueous environments where indirect radical effects dominate, gamma irradiation in the dry state can induce direct-action cross-linking between the TriRBD protein and the dense Tween-80 interface [50]. This process likely yields a particulate-like nano-complex upon dissolution, which is more efficiently internalized by antigen-presenting cells compared to soluble antigens [51,52]. Furthermore, the higher concentration of Tween-80 in the SqT formulation (1.0%) compared to SqTS (0.5%) appears to provide superior radioprotection, possibly by shielding critical epitopes from direct radiative damage, a phenomenon consistent with the established role of polysorbates in stabilizing biopharmaceuticals [53]. In contrast, the presence of the more hydrophobic Span-85 in the SqTS formulation may disrupt this interfacial uniformity in the solid state, leading to sub-optimal antigen protection and a less balanced immune profile following irradiation. Specially, Irradiated SqTS shifted toward a Th2 (IgG1) response over time, whereas SqT remained balanced. This suggests that the chemical environment of SqTS promotes a distinct type of radiation-induced protein modification under radiation, ultimately steering the immune system toward a less balanced, more allergic-type (Th2) pathway. Nevertheless, the precise biophysical impact of gamma irradiation on the reconstituted TriRBD-SqT complex remains a working hypothesis. Detailed physicochemical evaluations—including post-dissolution particle size distribution, subunit stability, secondary structure, and antigen release kinetics—alongside a direct comparison of SqTS versus SqT in intramuscular injection with antigen, will be conducted in future work. While the dry MAP format shows promising potential for enhanced stability, with physical integrity verified up to 20 months [9], functional stability assays and immunogenicity evaluations of adjuvanted MAPs across storage conditions will be a follow-up longitudinal study.

Overall, this SqT-adjuvanted MAP platform presents key manufacturing, economic, and regulatory benefits for pandemic preparedness. Continuous roll-to-roll micromolding dramatically reduces fabrication time to 8 min per patch up to 63,000 MAPs per line a day (Raphas Co., Ltd.) compared to >12 h for traditional casting [54]. Furthermore, terminal gamma irradiation ensures sterility without full aseptic processing suites, while solid-state stability minimizes cold-chain requirements.

Despite the promising results regarding the magnitude and durability of the antibody response, this study has several limitations that should be addressed in future research. First, while we observed a significant increase in TriRBD-specific IgG endpoint titers (EPT), we did not perform virus neutralization tests (VNT) or pseudovirus neutralization assays. Although total IgG levels often correlate with protective immunity, the ability of these antibodies to functionally block viral entry—particularly against highly mutated variants like Omicron—remains to be confirmed. Future studies should include neutralization assays to determine the quality and functional potency of the induced antibodies. Second, our analysis focused primarily on the humoral immune response (IgG subclasses). We did not evaluate cell-mediated immune responses, such as CD4+ and CD8+ T cell activation or cytokine profiling (e.g., IFN-γ, IL-4, IL-17). Characterizing functional T cell responses—including intracellular cytokine staining of recall-stimulated splenocytes alongside IgG1/IgG2a ratio—will be essential in follow-up studies to fully understand the vaccine’s protective breadth, particularly given the role of cellular immunity in preventing severe disease and cross-variant protection. Third, the protective efficacy of the MAP-TriRBD formulation was not validated through live virus challenge experiments in animal models (e.g., hACE2 transgenic mice or hamsters). Although our prior work demonstrated strong positive correlation between total anti-S1 IgG EPT, surrogate virus neutralization tests (sVNT) based on ACE2–trimer S binding inhibition, and micro-neutralization assays [28], high antibody titers alone do not guarantee full protection. Live-virus challenge studies remain essential to confirm whether this platform effectively reduces pulmonary viral load and prevents clinical symptoms or transmission. Finally, the relatively lower immunogenicity observed against the Omicron BA.1 S1 antigen highlights the challenge of overcoming significant antigenic distance. Although the SqT adjuvant improved the response, further optimization of the adjuvant concentration or the inclusion of more contemporary variant sequences (e.g., JN.1 or KP.2) may be required to ensure high-level protection against future evolving strains.

In summary, the combination of a trivalent RBD antigen with the SqT adjuvant in a microneedle format provides a potent and versatile tool against COVID-19 variants. While the Omicron BA.1 response was lower than the parental strain due to significant antigenic distance, the inclusion of SqT successfully bridged this gap by broadening the antibody repertoire. Future iterations may incorporate updated variant sequences to further enhance cross-reactivity.

## 5. Conclusions

This study demonstrates that the SARS-CoV-2 TriRBD vaccine—a chimeric trivalent recombinant protein incorporating Delta, Omicron BA.1, and XBB.1 RBDs, delivered via a dissolving microneedle array patch (MAP)—offers a superior and durable immunization strategy against evolving SARS-CoV-2 variants. Compared to traditional intramuscular administration, MAP delivery elicited significantly higher antibody titers that remained robust for up to 52 weeks. When formulated with a SqT adjuvant (Squalene/Tween-80) and sterilized via gamma irradiation, the MAP platform generated a balanced Th1/Th2 immune response and broadened antibody protection compared to SqTS adjuvant (Squalene/Tween-80/Span85, similar to AddaVax and MF59). Overall, this combination of broad immunity, long-term durability, safety, and thermostability establishes the microneedle platform as a promising tool against current variants and future pandemic threats.

## Figures and Tables

**Table 1:** Draize Scales for Scoring Primary Skin Irritation.

Erythema and Eschar Formation	Value	Edema Formation
No erythema	0	No edema
Very slight erythema (barely perceptible)	1	Very slight edema (barely perceptible)
Well-defibed erythema	2	Slight edema (edges of area well defined by definite raising)
Moderate-to-severe erythema	3	Moderate edema (raised approximately 1 mm)
Severe erythema (beet redness) to slight eschar formations (Injures in depth)	4	Severe edema (raised >1 mm and extending beyond the exposed area)

**Table 2:** Primary Irritation Index (PII).

	Values Referenced to		Sum	PII
	Erythema	Edema		
Empty MAP	1	0.8	1.8	0.46
MAP.SqTS	1.5	0.8	2.3	0.58

## Data Availability

The data sets used and analyzed during the current study are available from the corresponding author upon reasonable request.

## Supplementary Materials

The following supporting information can be downloaded at: https://www.mdpi.com/article/10.3390/pharmaceutics18101236/s1, Figure S1. Macroscopic observations of skin changes in six rabbits. Figure S2. Characterization of squalene oil-in-water emulsion adjuvants.
